# Supplementary material for: A UMLS-based spell checker for natural language processing in vaccine safety
Source: BMC Med Inform Decis Mak. 2007 Feb 12;7:3. doi: 10.1186/1472-6947-7-3 (PMC1805499; doi:10.1186/1472-6947-7-3)
Supplement: Additional file 13 — RAP application directory. Sets up directory for RAP (RDF application for PHP) source code [file 1472-6947-7-3-S13.gz › rap/doc/phpdoc/__filesource/fsource_rdql__rdqlRdqlMemEngine.php.html]

xml version="1.0" encoding="iso-8859-1"?


File Source for RdqlMemEngine.php


# Source for file RdqlMemEngine.php

Documentation is available at RdqlMemEngine.php

```
1. <?php

3. // ----------------------------------------------------------------------------------
4. // Class: RdqlMemEngine
5. // ----------------------------------------------------------------------------------


9. /**
10. * This class performes as RDQL query on a MemModel.
11. *
12. * Provided an rdql query parsed into an array of php variables and constraints
13. * at first the engine searches for tuples matching all patterns from the WHERE clause
14. * of the given RDQL query. Then the query result set is filtered with evaluated
15. * boolean expressions from the AND clause of the given RDQL query.
16. *
17. *
18. * <BR><BR>History:<UL>
19. * <LI>09-08-2004                 : findTriplesMatchingPattern() rewritten to only use the $model->find()
20. *                                   function for accessing the model. Changed the method of comparing literals to literals->equals
21. *                                    according to actual specification
22. * <LI>05-12-2004                 : Bug in the handling of empty Literals fixed.</LI>
23. * <LI>03-17-2004                 : Function findTuplesMatchingOnePattern()
24. *                                   pass-by-reference bug fixed</LI>
25. * <LI>08-29-2003                : Function filterTuples(): some bugs fixed:
26. *                                 - strEqExpr with NE operator
27. *                                 - regExExpr combined with other expr. and negation (!)
28. *                                   e.g. !(?x ~~ "/sth/" && ?x > 5)
29. * <LI>07-27-2003                : First version of this class</LI>
30. *
31. * @version  V0.9.1
32. * @author   Radoslaw Oldakowski <radol@gmx.de>
33. *
34. * @package rdql
35. * @access public
36. */

38. Class RdqlMemEngine extends RdqlEngine {


41. /**
42. * Parsed query variables and constraints.
43. *
44. * @var     array   ['selectVars'][] = ?VARNAME
45. *                   ['sources'][] = URI
46. *                   ['patterns'][]['subject']['value'] = VARorURI
47. *                                 ['predicate']['value'] = VARorURI
48. *                                 ['object']['value'] = VARorURIorLiterl
49. *                                           ['is_literal'] = boolean
50. *                                           ['l_lang'] = string
51. *                                           ['l_dtype'] = string
52. *                   ['filters'][]['string'] = string
53. *                                ['evalFilterStr'] = string
54. *                                ['reqexEqExprs'][]['var'] = ?VARNAME
55. *                                                  ['operator'] = (eq | ne)
56. *                                                  ['regex'] = string
57. *                                ['strEqExprs'][]['var'] = ?VARNAME
58. *                                                ['operator'] = (eq | ne)
59. *                                                ['value'] = string
60. *                                                ['value_type'] = ('variable' | 'URI' | 'Literal')
61. *                                                ['value_lang'] = string
62. *                                                ['value_dtype'] = string
63. *                                ['numExpr']['vars'][] = ?VARNAME
64. *                          ( [] stands for an integer index - 0..N )
65. * @access    private
66. */
67. var $parsedQuery;


70. /**
71. * Perform an RDQL Query on the given MemModel.
72. *
73. * @param   object  MemModel &$memModel
74. * @param   array   &$parsedQuery  (the same format as $this->parsedQuery)
75. * @param   boolean $returnNodes
76. * @return  array   [][?VARNAME] = object Node  (if $returnNodes = TRUE)
77. *       OR  array   [][?VARNAME] = string
78. *
79. * @access  public
80. */
81. function & queryModel(&$memModel, &$parsedQuery, $returnNodes = TRUE) {

83. $this->parsedQuery = $parsedQuery;

85. // find tuples matching all patterns
86. $res = $this->findTuplesMatchingAllPatterns($memModel);

88. // filter tuples
89. if (isset($parsedQuery['filters']))
90. $res = $this->filterTuples($res);

92. // select variables to be returned
93. $res = $this->selectVariables($res);

95. if(!$returnNodes)
96. return $this->toString($res);

98. return $res;
99. }


102. /**
103. * Find triples matching all patterns of an RDQL query and return an array
104. * with variables from all patterns and their corresponding values.
105. * The variable values returned are instances of object Node.
106. *
107. * @param   object  MemModel  &$memModel
108. * @return  array   [][?VARNAME] = object Node
109. *
110. * @access  private
111. */
112. function findTuplesMatchingAllPatterns(&$memModel) {

114. $resultSet = $this->findTuplesMatchingOnePattern($memModel, $this->parsedQuery['patterns'][0]);
115. for ($i=1; $i<count($this->parsedQuery['patterns']); $i++) {
116. $rs = $this->findTuplesMatchingOnePattern($memModel, $this->parsedQuery['patterns'][$i]);
117. $resultSet = $this->joinTuples($resultSet, $rs);
118. }
119. return $resultSet;
120. }


123. /**
124. * Find tuples matching one pattern and return an array with pattern
125. * variables and their corresponding values (instances of object Node).
126. *
127. * @param   object  MemModel &$memModel
128. * @param   array  &$pattern ['subject']['value'] = VARorURI
129. *                            ['predicate']['value'] = VARorURI
130. *                            ['object']['value'] = VARorURIorLiterl
131. *                                      ['is_literal'] = boolean
132. *                                      ['l_lang'] = string
133. *                                      ['l_dtype'] = string
134. * @return  array   [][?VARNAME] = object Node
135. *
136. * @access  private
137. */
138. function findTuplesMatchingOnePattern(&$memModel, &$pattern) {

140. $resultSet = array();
141. $i = 0;
142. // parameters to be passed to the method findTriplesMatchingPattern
143. foreach ($pattern as $key => $v) {
144. if ($v['value'] && $v['value']{0} == '?') {
145. if ($key == 'object') {
146. $param['object']['is_a'] = 'ANY';
147. $param['object']['string'] = 'ANY';
148. $param['object']['lang'] = NULL;
149. $param['object']['dtype'] = NULL;
150. } else
151. $param[$key] = 'ANY';
152. $var[$i]['key'] = $key;
153. $var[$i++]['val'] = $v['value'];
154. }else
155. if (isset($v['is_literal'])) {
156. $param[$key]['is_a'] = 'Literal';
157. $param[$key]['string'] = $v['value'];
158. $param[$key]['lang'] = $v['l_lang'];
159. $param[$key]['dtype'] = $v['l_dtype'];
160. }else{
161. if ($key == 'object') {
162. $param[$key]['is_a'] = 'Resource';
163. $param[$key]['string'] = $v['value'];
164. $param[$key]['lang'] = NULL;
165. $param[$key]['dtype'] = NULL;
166. }else
167. $param[$key] = $v['value'];
168. }
169. }

171. // find pattern internal bindings e.g. (?x, ?z, ?x)
172. $intBindings = NULL;
173. for ($i=0; $i<count($var); $i++)
174. foreach($var as $n => $v)
175. if ($i != $n && $var[$i]['val'] == $v['val'])
176. $intBindings[] = $var[$i]['key'];

178. // find triples of the $memModel matching $pattern
179. $resModel = $this->findTriplesMatchingPattern($memModel, $param['subject'],
180. $param['predicate'],
181. $param['object']['is_a'],
182. $param['object']['string'],
183. $param['object']['lang'],
184. $param['object']['dtype'],
185. $intBindings);

187. // set values of the pattern variables to be returned
188. if ($pattern['subject']['value']{0} == '?') {
189. $n = 0;
190. foreach ($resModel->triples as $triple)
191. $resultSet[$n++][$pattern['subject']['value']] = $triple->subj;
192. }
193. if ($pattern['predicate']['value']{0} == '?') {
194. $n = 0;
195. foreach ($resModel->triples as $triple)
196. $resultSet[$n++][$pattern['predicate']['value']] = $triple->pred;
197. }
198. if ($pattern['object']['value'] && $pattern['object']['value']{0} == '?') {
199. $n = 0;
200. foreach ($resModel->triples as $triple)
201. $resultSet[$n++][$pattern['object']['value']] = $triple->obj;
202. }
203. return $resultSet;
204. }


207. /**
208. * Search in $memModel for triples matching one pattern from the WHERE clause.
209. * 'ANY' input for $subjLabel..$objLabel, $obj_is will match anything.
210. * NULL input for $objDtype will only match obj->dtype = NULL
211. * NULL input for $objLanguage will match obj->lang = NULL or anything if a
212. * literal is datatyped (except for XMLLiterals and plain literals)
213. * This method also checks internal bindings if provided.
214. *
215. * @param   object MemModel $memModel
216. * @param   string $subjLabel
217. * @param   string $predLabel
218. * @param   string $objLabel
219. * @param   string $obj_is
220. * @param   string $objLanguage
221. * @param   string $objDtype
222. * @param   array  $intBindings [] = string
223. * @return  object MemModel
224. * @access    private
225. */
226. function findTriplesMatchingPattern(&$memModel, $subjLabel, $predLabel, $obj_is,
227. $objLabel, $objLang, $objDtype, &$intBindings) {

229. $res = new MemModel();

231. if($memModel->isEmpty())
232. return $res;

234. if ($subjLabel=='ANY')
235. {
236. $subj=NULL;
237. } else
238. {
239. $subj=new Resource($subjLabel);
240. };
241. if ($predLabel=='ANY')
242. {
243. $pred=NULL;
244. } else
245. {
246. $pred=new Resource($predLabel);
247. };

249. if ($objLabel=='ANY')
250. {
251. $obj=NULL;
252. } else
253. {
254. if ($obj_is == 'Literal')
255. {
256. $obj=new Literal($objLabel);
257. $obj->setDatatype($objDtype);
258. $obj->setLanguage($objLang);
259. } else {
260. $obj=new Resource($objLabel);
261. }
262. };

264. $res=$memModel->find($subj,$pred,$obj);

266. if ($intBindings)
267. foreach ($res->triples as $triple)
268. {
269. if (!$this->_checkIntBindings($triple, $intBindings))
270. {
271. $res->remove($triple);
272. }
273. }

275. return $res;
276. }


279. /**
280. * Perform an SQL-like inner join on two resultSets.
281. *
282. * @param   array   &$finalRes [][?VARNAME] = object Node
283. * @param   array   &$res      [][?VARNAME] = object Node
284. * @return  array              [][?VARNAME] = object Node
285. *
286. * @access  private
287. */
288. function joinTuples(&$finalRes, &$res) {

290. if (count($finalRes) == 0 || count($res) == 0)
291. return array();

293. // find joint variables and new variables to be added to $finalRes
294. $jointVars = array();
295. $newVars = array();
296. foreach ($res[0] as $varname => $node) {
297. if (array_key_exists($varname, $finalRes[0]))
298. $jointVars[] = $varname;
299. else
300. $newVars[] = $varname;
301. }

303. // eliminate rows of $finalRes in which the values of $jointVars do not have
304. // a corresponding row in $res.
305. foreach ($finalRes as $n => $fRes) {
306. foreach ($res as $i => $r) {
307. $ok = TRUE;
308. foreach ($jointVars as $j_varname)
309. if ($r[$j_varname] != $fRes[$j_varname]) {
310. $ok = FALSE;
311. break;
312. }
313. if ($ok)
314. break;
315. }
316. if (!$ok)
317. unset($finalRes[$n]);
318. }

320. // join $res and $finalRes
321. $joinedRes = array();
322. foreach ($res as $i => $r) {
323. foreach ($finalRes as $n => $fRes) {
324. $ok = TRUE;
325. foreach ($jointVars as $j_varname)
326. if ($r[$j_varname] != $fRes[$j_varname]) {
327. $ok = FALSE;
328. break;
329. }
330. if ($ok) {
331. $joinedRow = $finalRes[$n];
332. foreach($newVars as $n_varname)
333. $joinedRow[$n_varname] = $r[$n_varname];
334. $joinedRes[] = $joinedRow;
335. }
336. }
337. }

339. return $joinedRes;
340. }


343. /**
344. * Filter the result-set of query variables by evaluating each filter from the
345. * AND clause of the RDQL query.
346. *
347. * @param   array  &$finalRes  [][?VARNAME] = object Node
348. * @return  array  [][?VARNAME] = object Node
349. * @access    private
350. */
351. function filterTuples(&$finalRes) {

353. foreach ($this->parsedQuery['filters'] as $filter) {

355. foreach ($finalRes as $n => $fRes) {
356. $evalFilterStr = $filter['evalFilterStr'];

358. // evaluate regex equality expressions of each filter
359. foreach ($filter['regexEqExprs'] as $i => $expr) {

361. preg_match($expr['regex'], $fRes[$expr['var']]->getLabel(), $match);
362. $op = substr($expr['operator'], 0,1);
363. if (($op != '!' && !isset($match[0])) || ($op == '!' && isset($match[0])))
364. $evalFilterStr = str_replace("##RegEx_$i##", 'FALSE', $evalFilterStr);
365. else
366. $evalFilterStr = str_replace("##RegEx_$i##", 'TRUE', $evalFilterStr);

368. }

370. // evaluate string equality expressions
371. foreach ($filter['strEqExprs'] as $i => $expr) {

373. $exprBoolVal = 'FALSE';

375. switch ($expr['value_type']) {

377. case 'variable':
378. if (($fRes[$expr['var']] == $fRes[$expr['value']] && $expr['operator'] == 'eq') ||
379. ($fRes[$expr['var']] != $fRes[$expr['value']] && $expr['operator'] == 'ne'))
380. $exprBoolVal = 'TRUE';
381. break;

383. case 'URI':

385. if (is_a($fRes[$expr['var']], 'Literal')) {
386. if ($expr['operator'] == 'ne')
387. $exprBoolVal = 'TRUE';
388. break;
389. }

391. if (($fRes[$expr['var']]->getLabel() == $expr['value'] && $expr['operator'] == 'eq') ||
392. ($fRes[$expr['var']]->getLabel() != $expr['value'] && $expr['operator'] == 'ne'))
393. $exprBoolVal = 'TRUE';
394. break;

396. case 'Literal':

398. if (!is_a($fRes[$expr['var']], 'Literal')) {
399. if ($expr['operator'] == 'ne')
400. $exprBoolVal = 'TRUE';
401. break;
402. }

404. $filterLiteral= new Literal($expr['value'],$expr['value_lang']);
405. $filterLiteral->setDatatype($expr['value_dtype']);

407. $equal=$fRes[$expr['var']]->equals($filterLiteral);
408. /*                 if ($fRes[$expr['var']]->getLabel() == $expr['value'] &&
409. $fRes[$expr['var']]->getDatatype() == $expr['value_dtype']) {

411. $equal = TRUE;

413. // Lang tags only differentiate literals in rdf:XMLLiterals and plain literals.
414. // Therefore if a literal is datatyped ignore the language tag.
415. if ((($expr['value_dtype'] == NULL) ||
416. ($expr['value_dtype'] == 'http://www.w3.org/1999/02/22-rdf-syntax-ns#XMLLiteral') ||
417. ($expr['value_dtype'] == 'http://www.w3.org/2001/XMLSchema#string')) &&
418. (($fRes[$expr['var']]->getLanguage() != $expr['value_lang'])))

420. $equal = FALSE;
421. }else
422. $equal = FALSE;
423. */
424. if (($equal && $expr['operator'] == 'eq') ||
425. (!$equal && $expr['operator'] == 'ne'))
426. $exprBoolVal = 'TRUE';
427. else
428. $exprBoolVal = 'FALSE';

430. }
431. $evalFilterStr = str_replace("##strEqExpr_$i##", $exprBoolVal, $evalFilterStr);
432. }

434. // evaluate numerical expressions
435. foreach ($filter['numExprVars'] as $varName) {
436. $varValue = "'" .$fRes[$varName]->getLabel() ."'";
437. $evalFilterStr = str_replace($varName, $varValue, $evalFilterStr);
438. }

440. eval("\$filterBoolVal = $evalFilterStr; \$eval_filter_ok = TRUE;");
441. if (!isset($eval_filter_ok))
442. trigger_error(RDQL_AND_ERR ."'" .htmlspecialchars($filter['string']) ."'", E_USER_ERROR);

444. if (!$filterBoolVal)
445. unset($finalRes[$n]);
446. }
447. }

449. return $finalRes;
450. }


453. /**
454. * Remove all conditional variables from the result-set and leave only variables
455. * specified in the SELECT clause of the RDQL query.
456. *
457. * @param   array  &$finalRes  [][?VARNAME] = object Node
458. * @return  array  [][?VARNAME] = object Node
459. * @access    private
460. */
461. function selectVariables(&$finalRes) {

463. // if nothing has been found return only one row of $finalRes
464. // with select variables having empty values
465. if (count($finalRes) == 0) {
466. foreach ($this->parsedQuery['selectVars'] as $selectVar)
467. $finalRes[0][$selectVar] = NULL;
468. return $finalRes;
469. }

471. // return only selectVars in the same order as given in the RDQL query
472. // and reindex $finalRes.
473. $n = 0;
474. foreach($finalRes as $key => $val) {
475. foreach ($this->parsedQuery['selectVars'] as $selectVar)
476. $resultSet[$n][$selectVar] = $val[$selectVar];
477. unset($finalRes[$key]);
478. ++$n;
479. }

481. return $resultSet;
482. }


485. /**
486. * Convert the variable values of $finalRes from objects to their string serialization.
487. *
488. * @param   array  &$finalRes  [][?VARNAME] = object Node
489. * @return  array  [][?VARNAME] = string
490. * @access    private
491. */
492. function toString(&$finalRes) {

494. foreach ($finalRes as $n => $tuple)
495. foreach ($tuple as $varname => $node) {
496. if (is_a($node, 'Resource'))
497. $res[$n][$varname] = '<' .$node->getLabel() .'>';
498. elseif (is_a($node, 'Literal')) {
499. $res[$n][$varname] = '"' .$node->getLabel() .'"';
500. if ($node->getLanguage())
501. $res[$n][$varname] .= ' (xml:lang="' .$node->getLanguage() .'")';
502. if ($node->getDatatype())
503. $res[$n][$varname] .= ' (rdf:datatype="' .$node->getDatatype() .'")';
504. }else
505. $res[$n][$varname] = $node;
506. }
507. return $res;
508. }


511. /**
512. * Check if the given triple meets pattern internal bindings
513. * e.g. (?x, ?z, ?x) ==> statement subject must be identical with the statement object
514. *
515. * @param   object statement &$triple
516. * @param   array  &$intBindings [] = string
517. * @return  boolean
518. * @access    private
519. */
520. function _checkIntBindings (&$triple, &$intBindings) {

522. if (in_array('subject', $intBindings)) {
523. if (in_array('predicate', $intBindings))
524. if ($triple->subj != $triple->pred)
525. return FALSE;
526. if (in_array('object', $intBindings)) {
527. if (is_a($triple->obj, 'Literal'))
528. return FALSE;
529. elseif ($triple->subj != $triple->obj)
530. return FALSE;
531. }
532. return TRUE;
533. }
534. if (in_array('predicate', $intBindings)) {
535. if (is_a($triple->obj, 'Literal'))
536. return FALSE;
537. elseif ($triple->pred != $triple->obj)
538. return FALSE;
539. return TRUE;
540. }
541. }


544. /**
545. * Check if the lang and dtype of the passed object Literal are equal $lang and $dtype
546. * !!! Language only differentiates literals in rdf:XMLLiterals and plain literals (xsd:string).
547. * !!! Therefore if a literal is datatyped ignore the language.
548. *
549. * @param  object  Literal $literal
550. * @param  string  $dtype1
551. * @param  string  $dtype2
552. * @return boolean
553. * @access private
554. */
555. function _equalsLangDtype ($literal, $lang, $dtype) {

557. if ($dtype == $literal->getDatatype()) {
558. if (($dtype == NULL ||
559. $dtype == 'http://www.w3.org/2001/XMLSchema#string' ||
560. $dtype == 'http://www.w3.org/1999/02/22-rdf-syntax-ns#XMLLiteral') &&
561. ($lang != $literal->getLanguage()))
562. return FALSE;
563. return TRUE;
564. }
565. return FALSE;
566. }


569. } // end: Class RdqlMemEngine

571. ?>
```

Documentation generated on Thu, 7 Jul 2005 13:43:15 +0200 by phpDocumentor 1.3.0RC3
